# Phacoemulsification and Visco-synechiolysis With or Without Trabeculectomy Following Initial Management of Acute Primary Angle Closure: A Comparative Study

**DOI:** 10.18502/jovr.v20.15048

**Published:** 2025-05-19

**Authors:** Mahdi Sharifzadeh Kermani, Mina Haj-mohammad Karimi, Ali Sharifi, Mahla Shadravan, Arash Daneshtalab, Amin Zand

**Affiliations:** Clinical Research Development Unit, Shafa Hospital, Kerman University of Medical Sciences, Kerman, Iran

**Keywords:** Acute Primary Angle Closure, Glaucoma, Peripheral Anterior Synechiae, Phacoemulsification, Trabeculectomy, Visco-synechiolysis

## Abstract

**Purpose:**

To assess the effects of phacoemulsification, visco-synechiolysis, and trabeculectomy on eyes with a recent history of acute primary angle closure (APAC).

**Methods:**

In this prospective nonrandomized study, we enrolled patients with cataracts, peripheral anterior synechiae (PAS), and a history of APAC attack managed with medications and laser peripheral iridotomy (LPI) within the past six weeks. Patients without signs of glaucomatous optic neuropathy (GON) underwent phacoemulsification and visco-synechiolysis (PV group). Trabeculectomy was added to this procedure for cases with signs of underlying chronic GON (PVT group). We evaluated best-corrected visual acuity (BCVA), intraocular pressure (IOP), angle opening, PAS extension, and adverse events at baseline and six months postoperatively.

**Results:**

The PV and PVT groups comprised 8 and 12 eyes, respectively. At month six, both groups showed significant improvement in BCVA, reduced IOP, and increased Shaffer grading scores compared to baseline (all *P*s < 0.05). Extensive PAS ( ≥ 180º) significantly decreased at month six in both the PV (*P* = 0.008) and PVT (*P* = 0.002) groups compared to baseline. However, its prevalence did not significantly differ between the two groups at baseline (*P* = 0.288) or six months after surgery (*P* = 0.881). At month six, IOP was significantly lower in the PVT group than the PV group (10.83 ± 1.40 vs 13.63 ± 2.07 mmHg, *P* = 0.002). Nevertheless, BCVA and Shaffer grading scores were not different between the two groups at this time point (*P* = 0.120, and *P* = 0.891, respectively). No serious complications were observed in any groups during the follow-ups.

**Conclusion:**

Patients with cataracts and a recent history of APAC without underlying chronic glaucoma may not receive additional trabeculectomy alongside lens extraction and synechiolysis.

##  INTRODUCTION

Acute primary angle closure (APAC) is characterized by a rapid increase in intraocular pressure (IOP) accompanied by a mid-dilated pupil.^[1,2,3]^ The primary objectives of initial treatment are twofold: reduction of IOP and alleviation of angle closure. While laser peripheral iridotomy (LPI) effectively treats angle closure, eyes undergoing this procedure remain susceptible to glaucoma progression.^[2,3,4,5]^ One hypothesis explaining glaucoma development in such cases involves the constriction of the angle and anterior chamber due to a thick crystalline lens.^[6,7,8]^ Consequently, lens extraction offers the potential to lower IOP, alleviate angle closure, and mitigate the risk of glaucoma progression in individuals with a history of APAC.^[9,10,11,12]^ Nevertheless, instances involving peripheral anterior synechiae (PAS) or indications of glaucomatous optic neuropathy (GON) demand additional surgical interventions alongside lens extraction.^[13]^ This study seeks to compare the outcomes of early phacoemulsification combined with visco-synechiolysis (VSL) for patients without GON or VSL + trabeculectomy for patients with underlying chronic GON. The subjects include eyes with PAS, cataracts, and a recent history of APAC attacks.

##  METHODS

This study adhered to the principles of the Declaration of Helsinki. The written consent was obtained from all participants, and the Ethics Committee of Kerman University of Medical Sciences, Kerman, Iran approved the study (Ethics code: IR.KMU.AH.REC.1400.296).

In this prospective and nonrandomized study, we enrolled 20 eyes of 20 patients. The patients were recruited from the eye emergency department and the glaucoma clinic of Shafa Hospital, Kerman, Iran, from May 2021 to April 2022.

Patients with PAS, medium to higher grades of cataracts,^[14]^ and a history of APAC attack management within six weeks of using both medications and LPI were included. APAC was confirmed when the following criteria were met: (1) experiencing at least two of the following symptoms: ocular pain, nausea and/or vomiting, and blurred vision; (2) IOP exceeding 25 mmHg along with at least three of the following signs: corneal epithelial edema, conjunctival injection, mid-dilated pupil, and shallow anterior chamber; and (3) evidence of occluded angle confirmed by gonioscopy. Angle was considered occluded if the posterior trabecular meshwork was not observable in at least three quadrants.^[15]^

Baseline and follow-up visits included assessing best-corrected visual acuity (BCVA), slit-lamp biomicroscopy, IOP measurement using Goldmann applanation tonometry, gonioscopy with and without indentation using a Zeiss-style 4-mirror goniolens, and fundus examination with a 90-diopter lens. The diagnosis of GON was based on features like optic nerve head cupping, visual field changes, or retinal nerve fiber layer (RNFL) loss in peripapillary optical coherence tomography (OCT). To account for the transient increase in mean RNFL thickness following an APAC attack due to edema, we performed OCT imaging at least two weeks after the initial management of the attack.^[16]^ This delay allowed for a more accurate assessment of the optic neuropathy. Additionally, we examined any available previous visual fields or peripapillary OCTs for each patient to enhance the precision of diagnosing the underlying chronic glaucoma. The exclusion criteria comprised age below 18 years, follow-up duration of less than six months, prior intraocular surgery or trauma, secondary causes of angle closure glaucoma, no signs of PAS in gonioscopy, previous treatment for APAC, corneal edema or opacity, clear lens or mild cataract, and inadequate compliance with anti-glaucoma eye drop instillation.

For all patients, APAC attacks were managed by intravenous mannitol followed by oral acetazolamide (1 gr/day), topical dorzolamide and brimonidine (twice daily), and betamethasone (four times daily). IOP was measured at 2 hours, 6 hours, and 1 day after attack initiation. Additionally, all participants underwent LPI on day one following the attack. Laser treatment commenced with a single 4-mJ pulse (Nd:YAG laser) after administering 2% pilocarpine; the power was escalated until achieving a 200 μ m patent opening, which was confirmed by fluid flush visualization. One glaucoma specialist (MSK) executed all laser procedures for all cases.

Attack relief was defined as an IOP < 25 mmHg (with or without medication) coupled with the abatement of acute IOP rise symptoms.^[15]^ Patients with cataracts were categorized into two groups: individuals without GON designated for phacoemulsification and VSL (PV group), and cases featuring underlying chronic GON signs who underwent phacoemulsification, VSL, and trabeculectomy (PVT group). A single surgeon (MSK) performed all surgeries using a standard phacoemulsification technique with one-piece hydrophobic acrylic IOL implantation (enVista, Bausch + Lomb). The incision site was separate from the trabeculectomy site and was located temporally on the cornea.

VSL was performed by injecting a viscoelastic gel into the peripheral anterior chamber without involving the angle structure. This was followed by synechiolysis under direct gonioscopic control using intraoperative goniolens. The procedure was completed by subsequent irrigation.^[17]^

**Table 1:** Baseline demographic and clinical characteristics of the surgical groups

	**PV group (** ***n*** ** = 8)**	**PVT group (** ***n*** ** = 12)**	***P*** **-value**
Age (yrs), mean ± SD	60.88 ± 11.64	61.75 ± 5.94	0.826
Female, *n* (%)	6 (75 %)	5 (41.67 %)	0.197
Vertical cup-to-disc ratio	0.47 ± 0.13	0.76 ± 0.25	0.006
n, number; PV, phacoemulsification and visco-synechiolysis; PVT, phacoemulsification, visco-synechiolysis, and trabeculectomy			

**Table 2:** Changes in clinical characteristics of the surgical groups at baseline and the final (month six) follow-up visit

	**Group**	**Baseline**	**Month six**	***P*** ** (within group)**
BCVA (logMAR), mean ± SD	PV group	1.16 ± 0.40	0.52 ± 0.38	0.012
	PVT group	1.25 ± 0.37	0.81 ± 0.40	0.003
	*P* (between groups)	0.606	0.120	
IOP (mmHg), mean ± SD	PV group	31.63 ± 11.22	13.63 ± 2.07	0.012
	PVT group	33.58 ± 8.08	10.83 ± 1.40	0.002
	*P* (between groups)	0.655	0.002	
Shaffer gonioscopy grading, score	PV group	1.13 ± 0.64	1.88 ± 0.64	0.014
	PVT group	0.58 ± 0.51	1.92 ± 0.67	0.002
	*P* (between groups)	0.067	0.891	
BCVA, best-corrected visual acuity; logMAR, logarithm of the minimum angle of resolution; IOP, intraocular pressure; PV, phacoemulsification and visco-synechiolysis, PVT, phacoemulsification, visco-synechiolysis, and trabeculectomy				

**Table 3:** Changes in PAS extension in the PV and PVT groups at baseline and the final (month six) follow-up visit

	**PAS extension (degree)**	**Baseline, ** ***n*** ** (%)**	**Month six, ** ***n*** ** (%)**	***P*** ** (within group)**
PV group (*n* = 8)	< 180º	6 (75)	7 (87.5)	0.008
	≥ 180º	2 (25)	1 (12.5)	
PVT group (*n* = 12)	< 180º	6 (50)	11 (91.67)	0.002
	≥ 180º	6 (50)	1 (8.33)	
*P* (between groups)		0.288	0.881	
n, number; PAS, peripheral anterior synechiae; PV, phacoemulsification and visco-synechiolysis; PVT, phacoemulsification, visco-synechiolysis, and trabeculectomy				

Trabeculectomy involved creating a fornix-based conjunctival flap and a limbus-based 4 × 4-mm scleral flap, under which mitomycin C 0.04%-soaked sponges were placed for 2 minutes. A 50 ml balanced saline solution was applied to rinse the surgical area, after which a peripheral iridectomy was performed. The scleral flap was closed with releasable 10-0 nylon sutures. Finally, the tenon capsule and conjunctiva were separately closed using 10-0 nylon sutures.^[18]^

Follow-up visits included comprehensive ophthalmic assessments, Goldmann applanation tonometry, and gonioscopy (with and without indentation) at baseline, day one, day seven, month one, and month six, all of which were conducted by a single observer (MHK). Besides, logMAR visual acuity was calculated based on BCVA measurements for statistical analyses. Angle evaluation was performed using the Shaffer grading system.^[19]^ PAS was defined as any abnormal adhesions of the iris to the angle at the level of the trabecular meshwork that could not be broken by indentation.^[15]^ Extensive PAS was defined as ≥ 180º PAS. Surgical complications were monitored during surgery and follow-up visits.

Postoperatively, all patients received topical ciprofloxacin four times daily for one week and betamethasone four times daily for two weeks, with gradual tapering over the subsequent month. Additional topical anti-glaucoma medication was prescribed if IOP exceeded 21 mmHg.

### Statistical Analysis 

Data were analyzed using SPSS version 24 (SPSS Inc., Chicago, IL, USA). Continuous variables were presented as mean ± SD and categorical variables as percentages. The *t*-test and chi-square test were employed to respectively compare continuous and categorical variables between the study groups. Within-group comparisons of preoperative and postoperative data were performed using the Wilcoxon signed-rank test. Statistical significance was set at *P* < 0.05.

##  RESULTS

This study was conducted on a total of 20 eyes of 20 patients, 55.0% of whom were female. The mean age of participants was 61.40 ± 8.40 years, and all of them had experienced an APAC attack within the past six weeks and exhibited PAS with at least a moderate cataract. The mean initial IOP before attack management was 45.15 ± 13.39 mmHg. A gonioscopy performed before LPI revealed closed or poorly visible angles due to corneal edema. Surgical interventions were conducted within six weeks following the attack. Baseline examination before surgery displayed a mean IOP of 32.80 ± 9.23 mmHg, with 15 (75.0%) patients exhibiting IOP levels exceeding 25 mmHg. The mean Shaffer gonioscopy grading score was 0.80 ± 0.62. Eight patients were assigned to the PV group, while the PVT group comprised 12 patients with GON. The baseline vertical cup-to-disc ratio was significantly higher in the PVT group (0.76 ± 0.25) compared to the PV group (0.47 ± 0.13, *P* = 0.006). Baseline demographic and clinical characteristics, including age, gender, BCVA, IOP, and Shaffer gonioscopy grading, were comparable in the two groups (all *P*s > 0.05). Further details of baseline demographic and clinical characteristics are summarized in Tables 1 and 2.

At the final follow-up visit (six months postoperatively), both the PV and PVT groups exhibited significant improvement in BCVA, IOP reduction, and higher Shaffer grading scores compared to baseline (all *P*s < 0.05). IOP was significantly lower in the PVT group than the PV group (10.83 ± 1.40 vs 13.63 ± 2.07 mmHg, *P* = 0.002) at month six. However, BCVA and Shaffer grading scores did not differ between the two groups at the end of the follow-up period (*P* = 0.120, and *P* = 0.891, respectively) [Table 2].

Table 3 provides an overview of PAS extension in the PV and PVT groups. The prevalence of extensive ( ≥ 180º) PAS did not significantly differ between these groups at baseline (*P* = 0.288) or six months after surgery (*P* = 0.881). However, extensive PAS notably decreased at month six in both the PV (*P* = 0.008) and PVT (*P* = 0.002) groups compared to baseline values.

Throughout the follow-up period, we did not observe any serious complications, including shallow/flat anterior chamber, hypotony (IOP < 4 mmHg), choroidal effusion, aqueous misdirection, bleb leakage, ischemia, or infectious endophthalmitis. Additionally, no patient experienced a recurrent attack, as all iridotomies remained patent.

##  DISCUSSION

Despite the effectiveness of LPI in preventing the progression of primary angle closure glaucoma in APAC-affected eyes, research has shown that certain eyes can still progress to glaucoma despite undergoing laser iridotomy.^[1,2,4,12,20,21]^ Hence, additional interventions in these cases become imperative to mitigate the risk of subsequent glaucoma development.

Previous studies have demonstrated that phacoemulsification alone can reduce IOP and create a deeper anterior chamber in PACG eyes. During phacoemulsification, the effusion of an ophthalmic viscoelastic device and saline in the anterior chamber may partially help lyse the PAS.^[22]^ However, in cases of PACG with PAS, the trabecular meshwork might remain obstructed by PAS post-lens extraction. In such scenarios, various techniques of VSL should be employed to break the PAS.^[13,17]^ Timely PAS lysis can halt the progression of irreversible structural alterations in the angle and prevent the advancement of resistant glaucoma stages. Hence, the combination of phacoemulsification and VSL seems promising for PACG patients with PAS.^[23,24]^

Our findings substantiate that early phacoemulsification, when combined with VSL, successfully opens the angle, diminishes PAS, and effectively controls IOP in patients with a recent history of APAC. Similarly, previous studies have reported that the combination of phacoemulsification and VSL effectively reopens the angle and reduces IOP in PACG patients.^[17,25,26]^ Moghimi et al also demonstrated that the combined approach had a greater effect on angle opening compared to standalone phacoemulsification, although there were no differences in long-term IOP reduction between the two groups.^[25]^

For individuals presenting with both PAS and GON, the combination of phacoemulsification/VSL with trabeculectomy could better reduce IOP and control glaucoma progression. Previous studies have indicated that while trabeculectomy remains a standard procedure for medically resistant glaucoma, combining it with phacoemulsification will reduce the success rate compared to standalone trabeculectomy.^[27,28]^ Furthermore, trabeculectomy is often associated with significant intra- and postoperative complications, including choroidal hemorrhage, prolonged hypotony due to excessive bleb filtration, and bleb-associated endophthalmitis.^[29]^ Our study found that the highest rate of IOP reduction was achieved in patients allocated to the PVT group, and this reduction was significantly greater than that in the PV group. However, the addition of trabeculectomy did not significantly impact angle opening or extensive PAS reduction. Moreover, no significant adverse events were observed across any groups, including those who underwent trabeculectomy, within the relatively short follow-up period. Tham et al demonstrated that in PACG eyes, the combined approach of phacoemulsification and trabeculectomy reduces IOP more than does standalone phacoemulsification.^[10,30]^ However, they noted that angle opening was superior in eyes undergoing standalone phacoemulsification. Furthermore, the combined approach was associated with a higher incidence of severe intra- and postoperative complications compared to phacoemulsification alone.

This study does have some limitations, including its nonrandomized nature and small sample size, which may have impacted the analyses. Moreover, the relatively short six-month follow-up period, combined with infrequent visits, could have led to incomplete documentation of some surgical failures or complications. It is essential to acknowledge that this limitation was due to the specific composition of the patient population, many of whom were referred to the glaucoma surgeon from distant areas, presenting logistical challenges for longer and more frequent follow-ups. Furthermore, our study did not document specific ocular characteristics such as visual field parameters, precise angle grading (e.g., by anterior segment OCT), PAS degree measurements, and bleb morphology. Therefore, there is a need for studies with larger sample sizes and extended and more frequent follow-ups.

In summary, our study demonstrated that the addition of trabeculectomy to combined phacoemulsification and VSL may not significantly alter the outcomes of angle opening and extensive ( ≥ 180º) PAS reduction in patients with cataracts and a recent history of APAC. Nevertheless, incorporating trabeculectomy into the procedure can yield a higher IOP reduction. Hence, patients with cataracts and a recent history of APAC, without underlying chronic glaucoma, might not get additional benefits from including trabeculectomy alongside lens extraction and synechiolysis.

##  Financial Support and Sponsorship 

None.

##  Conflicts of Interest

None.
